# Seventy-five mosses and liverworts found frozen with the late Neolithic Tyrolean Iceman: Origins, taphonomy and the Iceman’s last journey

**DOI:** 10.1371/journal.pone.0223752

**Published:** 2019-10-30

**Authors:** James H. Dickson, Klaus D. Oeggl, Werner Kofler, Wolfgang K. Hofbauer, Ronald Porley, Gordon P. Rothero, Alexandra Schmidl, Andreas G. Heiss

**Affiliations:** 1 Institute of Biodiversity, Animal Health & Comparative Medicine, University of Glasgow, Glasgow, Scotland, United Kingdom; 2 Institut für Botanik, University of Innsbruck, Innsbruck, Austria; 3 Franhofer Institut Bauphysik, Valley, Germany; 4 English Nature, Crookham Common, United Kingdom; Seoul National University College of Medicine, REPUBLIC OF KOREA

## Abstract

The Iceman site is unique in the bryology of the Quaternary. Only 21 bryophytes (mosses and liverworts) grow now in the immediate vicinity of the 5,300 year old Iceman discovery site at 3,210m above sea level in the Ötztal Alps, Italy. By contrast 75 or more species including at least ten liverworts were recovered as subfossils frozen in, on and around the Iceman from before, at and after his time. About two thirds of the species grow in the nival zone (above 3,000m above sea level) now while about one third do not. A large part of this third can be explained by the Iceman having both deliberately and inadvertently carried bryophytes during his last, fatal journey. Multivariate analyses (PCA, RDA) provide a variety of explanations for the arrivals of the bryophytes in the rocky hollow where the mummy was discovered. This is well into the nival zone of perennial snow and ice with a very sparse, non-woody flora and very low vegetation cover. Apart from the crucial anthropochory (extra-local plants), both hydrochory (local species) and zoochory (by wild game such as ibex of both local and extra-local species) have been important. Anemochory of mainly local species was of lesser importance and of extra-local species probably of little or no importance. The mosses *Neckera complanata* and several other ecologically similar species as well as a species of *Sphagnum* (bogmoss) strongly support the claim that the Iceman, took northwards up Schnalstal, South Tyrol, as the route of the last journey. A different species of bogmoss, taken from his colon is another indication the Iceman’s presence at low altitude south of Schnalstal during his last hours when he was first high up, low down and finally at over 3,000m.

## Introduction

**Fig 1:**
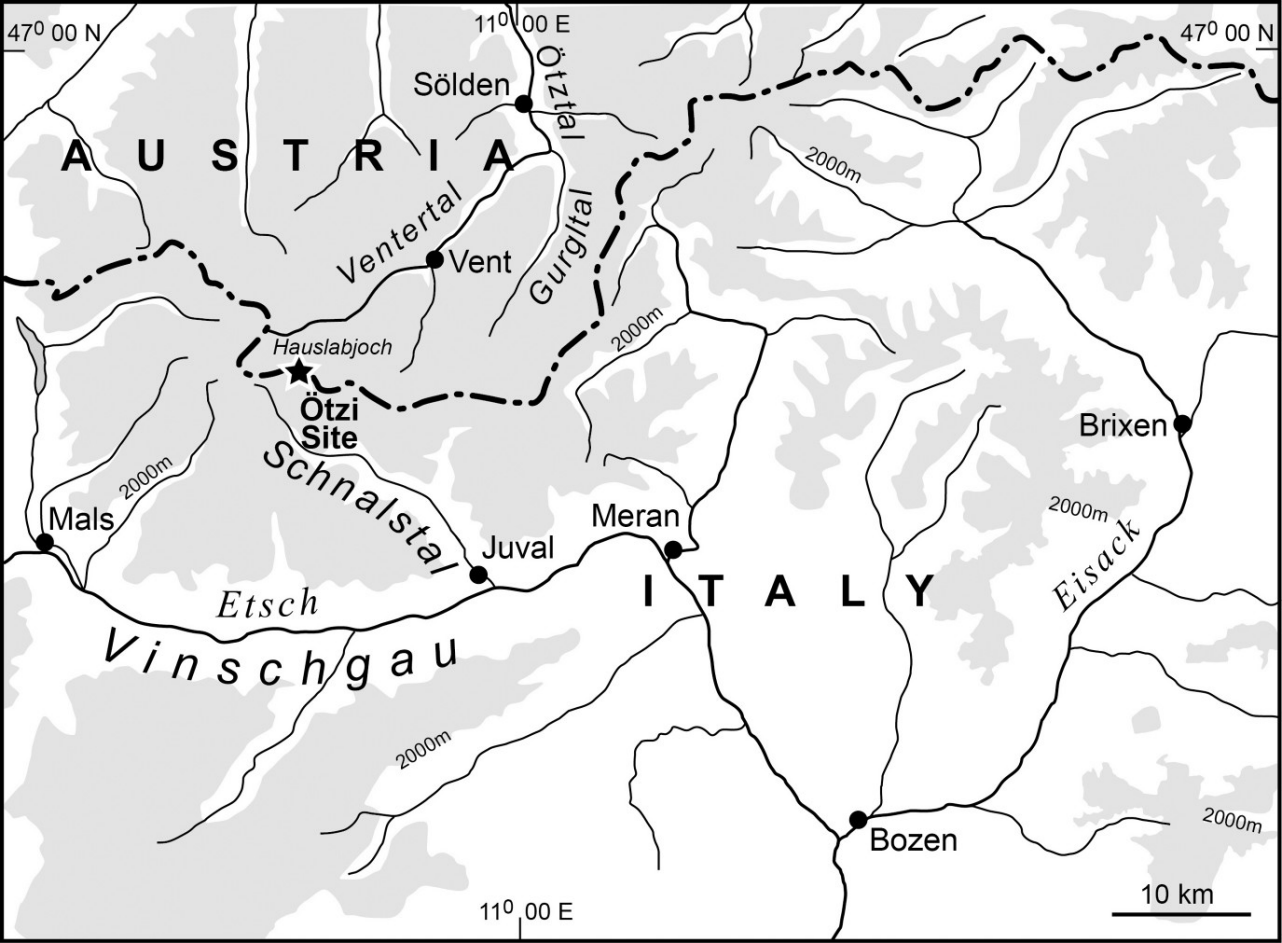
Location map. The star shows the Ötzi discovery site situated only 92m inside Italy.

When discovered in 1991 the mummified body of a man melting out of ice high in the Alps (3,210m above sea level) was a fully justified sensation worldwide (Fig 1). Nothing quite like it had been found previously. Soon nick-named “Ötzi” and superbly preserved though shrivelled, the man had been well clothed and shod. There was a diverse set of gear including a copper headed axe, a full set of archery equipment and a fire making kit in a belly bag. Much about Ötzi is controversial but not when he had lived which was about 5,200 to 5,300 years ago, as a large set of radiocarbon dates testify conclusively [1]. He had reached an age in the mid-forties and had suffered an array of ailments, such as atherosclerosis, gallstones and whipworms, as well as injuries both old (a frost bitten little left toe and broken ribs) and very fresh wounds. Right handed, he had a badly cut right palm inflicted perhaps 48 hours or less before the fatal arrow shot fired by a “South Alpine archer” [2]. This pierced his back under the left shoulder, punctured his scapula and ruptured the subclavicular artery. This was enough to ensure his speedy death by exsanguination [3, 4, 5]. The Iceman died with meat of alpine ibex and red deer in his gut [6]. His stomach contained a “remarkably high” proportion of fat [7]. From an early stage there has been the claim, much repeated, that Ötzi was a herdsman. There is strong scientific evidence that this was not so [8, 9, 10]. Ötzi’s stone disc with leather tassels shows marked parallels with modern game bird holders [11], another indication that the Iceman was a hunter. The copper ore for the axe head was of southern Tuscan origin [12], a long way to the south.

With the mummy there were many plant and fungal remains both microscopic (pollen, spores and diatoms) and macroscopic (seeds, cereals, leaves, wood, charcoal, bracket fungi and bryophytes). The detailed studies of these plants and fungi have produced an abundance of very informative data concerning life-style and diet [13, 14] as well as a reconstruction of the events during the last few days of a prehistoric man; this latter is without exact parallel in archaeology [15]. It is an outstanding outcome of archaeobotany. JHD was brought into the investigation to examine the bryophyte remains and this proved a productive task [5, 16, 17, 18, 19, 20, 21, 22].

**Fig 2:**
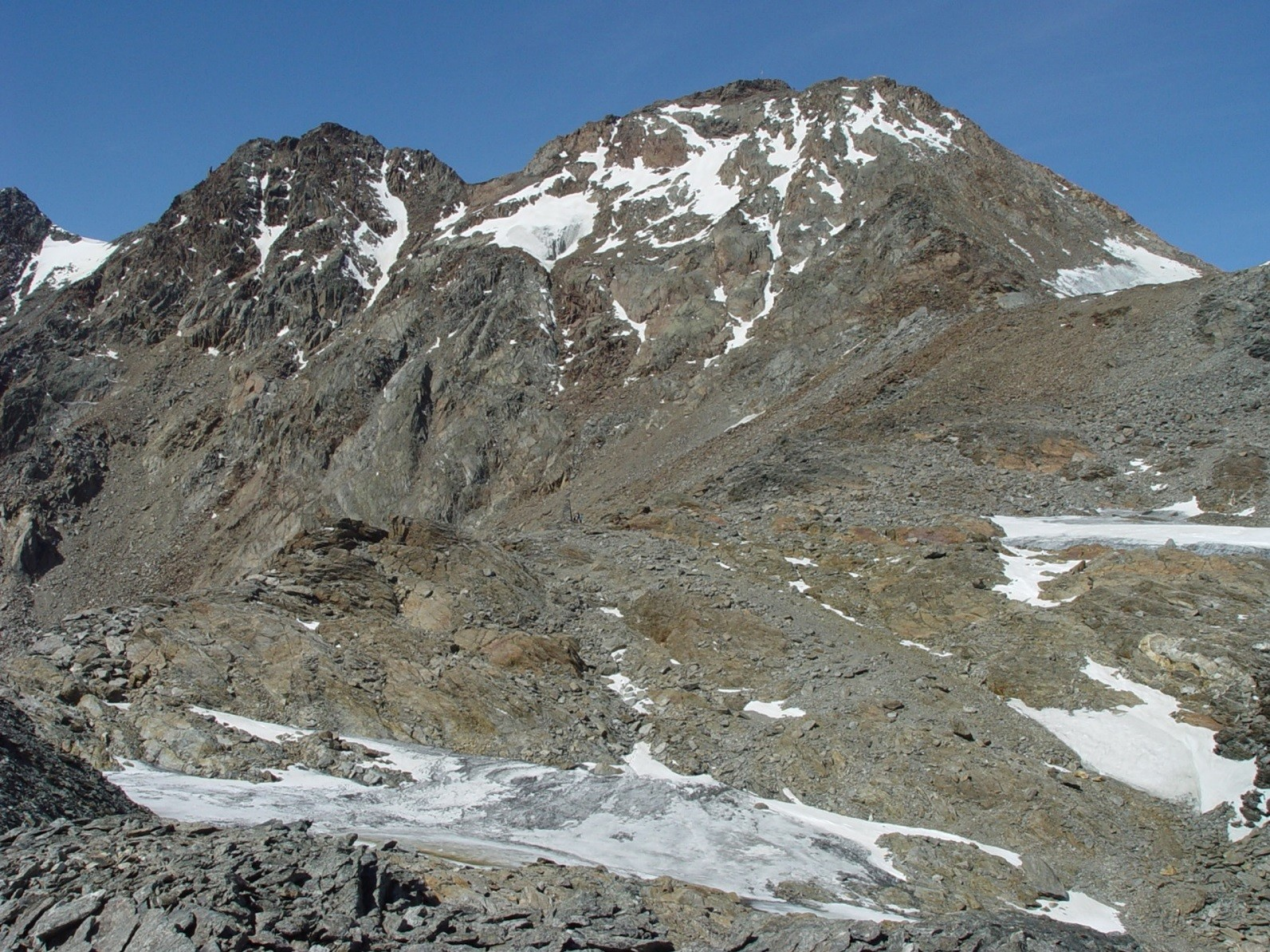
Looking down westwards to the Ötzi site. The monument, an obelisk, can be discerned just left of centre, September 2007. The Ötzi site was free of ice. All the ground is well above the nival zone upwards from 3,000m to the summit of Finailspitze at 3,514m.

**Fig 3:**
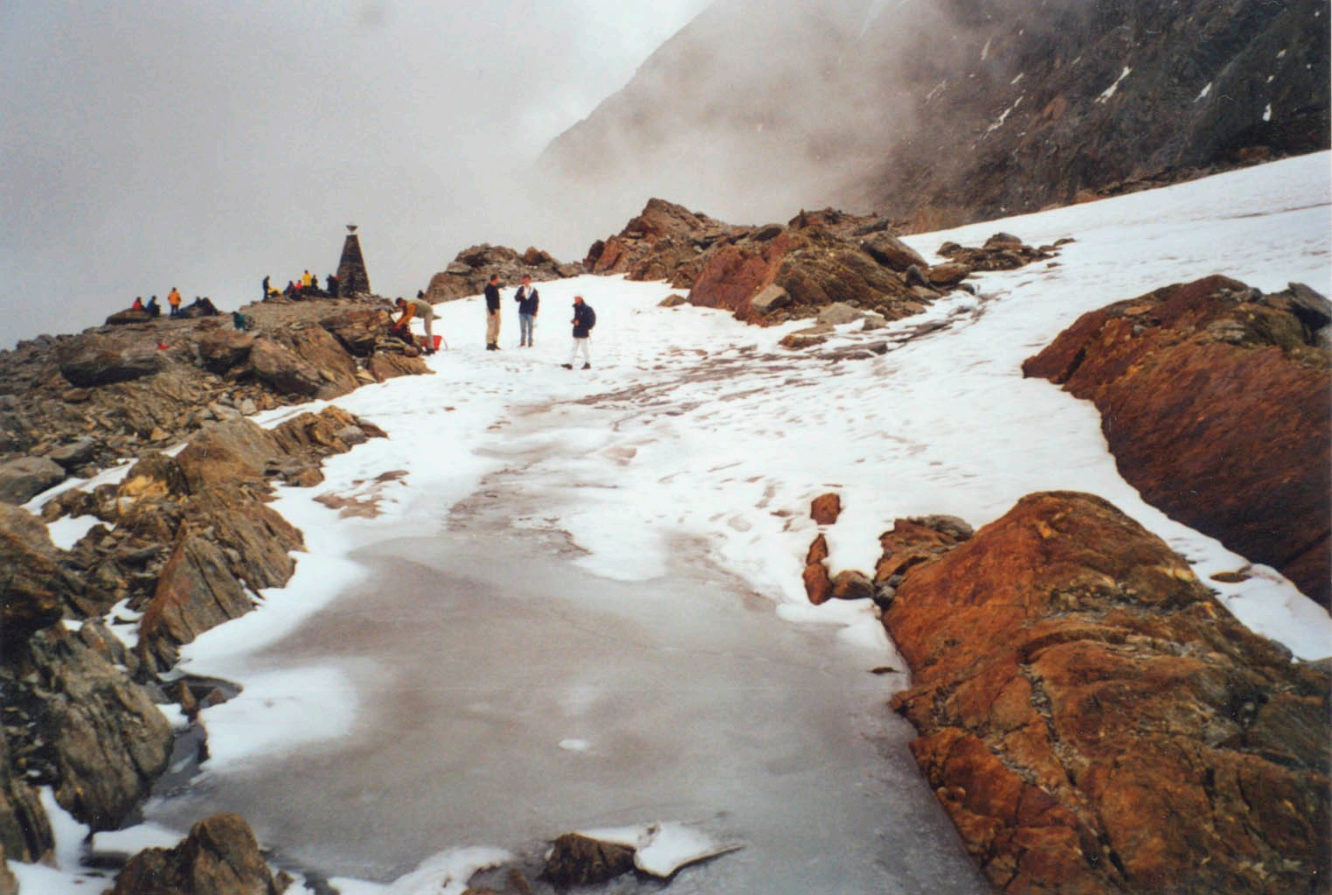
The Ötzi site still iced up, late August 2000. The footprints in the snow give an indication of scale. The meltwater goes to the Danube drainage.

The discovery site is located right at the top of the Tisenjoch pass near Hauslabjoch on the main Alpine ridge, restricted by steep rocky slopes to the south and a smooth descending glacier to the north (Figs 2 and 3). The topography of the pass area is rocky ridges and narrow gullies; in one of these the Iceman was discovered lying prone on a boulder. With the body all his gear and weapons were frozen in ice. However, the gully there acted as sediment trap which caught a complex find assemblage. Together with his equipment other plant/organic material was incorporated in the ice but also in the sediment of the gully. All the equipment and the sediment was recovered from the gully and analysed [14]. Here we refer to the plant remains retrieved from these ice and sediment samples, which encompass numerous vascular plants and bryophytes. By no means all of these plant remains were transported to the site by Ötzi, many of them were transported by natural vectors e.g. animals and water to the site.

The Iceman site is situated in the nival zone which is that vegetation zone at great enough altitude for the low temperatures to allow perennial snow and ice and a predominance of bare rock and scree [23]. With the dwarf shrub heaths and closed grasslands of the alpine zone below, the nival zone begins in Tyrol at about 3,000 m above sea level, as it does in Engadine, that part of Switzerland nearest Tyrol. Lacking dwarf shrubs, the total vegetation cover of the nival zone is very low and the total numbers of species of vascular plants, bryophytes and lichens are low because of the great exigencies of the environment. These numbers may be somewhat greater than realised especially for small plants like bryophytes simply because many mountains of great enough altitude are difficult of access for non-alpinist bryologists even in the short period when the snow cover and hostile conditions are minimal (mid August-mid September in Tyrol).

Leafy bryophyte stem fragments and detached leaves are known to be carried externally (ectozoochory) by large mammals such as sheep, roe deer, wild boar and dogs [24, 25, 26] and birds [27]. On one occasion WKH and JHD had the chance to see fragments of mosses and other plants adhering to sheep during the journey from Schnalstal to the high level pastures past the Iceman site in Austria. Such fragments can be carried internally (endozoochory) by mammals. This is vividly shown by the gut contents of the frozen mummies of the Siberian mammoths and humans including the Iceman [22]. Birds can carry moss fragments internally [28].

Dispersal by water (hydrochory) is too obvious to need amplification. Wind dispersal (anemochory) of bryophyte fragments of local species onto snowbeds and into hollows has been well demonstrated for treeless parts of Arctic Canada and elsewhere in North America [29, 30, 31]. There appears to be little data concerning wind dispersal of leafy bryophyte fragments over very large distances.

The plant remains retrieved from the ice show a wide distribution from valley bottoms to the summits. Some of them grow on the valley bottoms only, some thrive only at high altitudes. Here we aim to evaluate the way these bryophyte remains were deposited at the site by macro remain analyses, numerical methods and an extensive field survey contributing to the present chorology of the bryophyte subfossils. We have classified how the bryophytes found their way to the site according to their frequency in the samples. A special focus is laid on the low-altitude bryophytes, because they have the potential to contribute to the detailed reconstruction of the final ascent of Ötzi.

The full list of subfossil bryophytes recovered from the Iceman site is given in S1 Appendix.

The overall aim of the project was to appreciate to fullest extent the significance of the numerous anciently frozen Iceman bryophytes for bryology and archaeology. It has been substantially achieved.

## Methods

### Taphonomy

**Fig 4:**
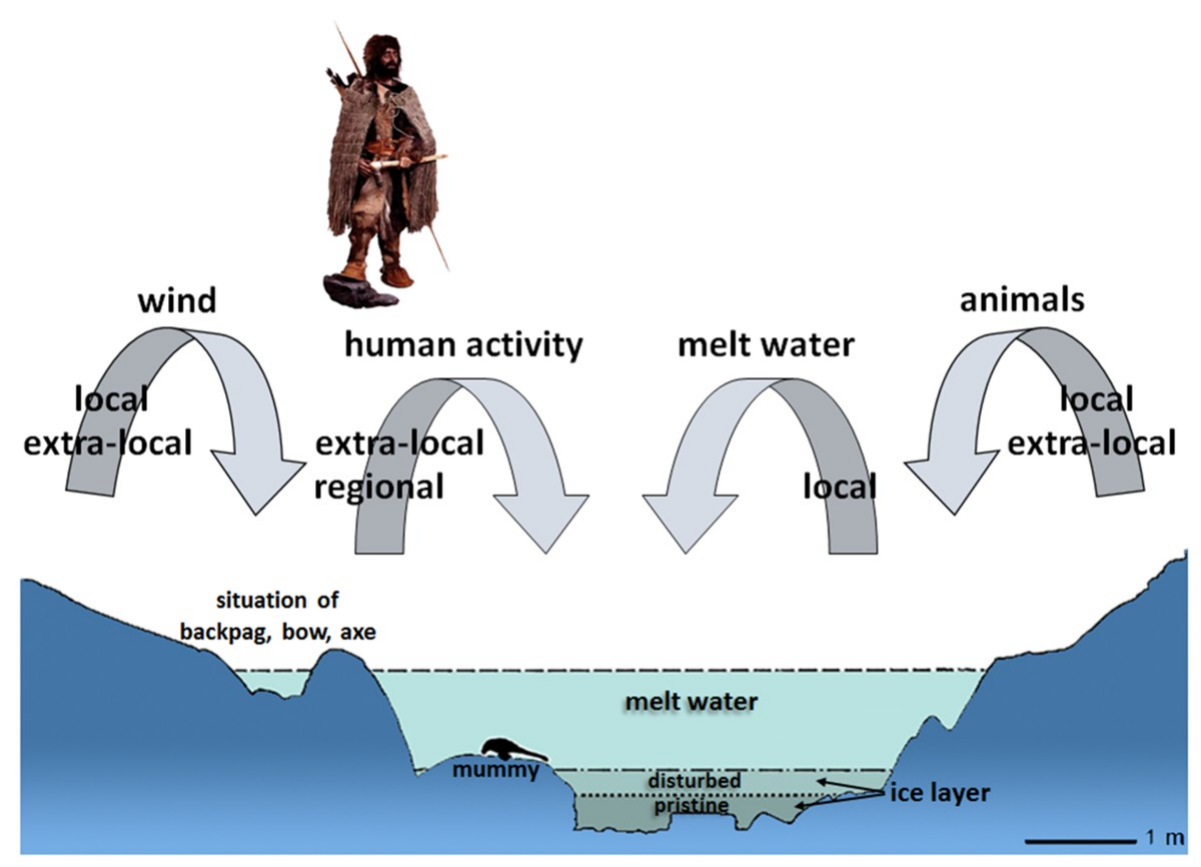
Taphonomic processes. These led to the deposition of flowering plant remains at the Ötzi discovery site according to Heiss and Oeggl [14].

A detailed understanding of the taphonomy is a crucial matter (Fig 4). How were the bryophyte fragments transported to the discovery site leading to preservation? The narrow, shallow rocky gully, in which the mummified corpse was discovered, is a sediment trap (Fig 4). The organic remains were transported either by melt water (hydrochory), wind (anemochory) or animal/human activity (zoochory/anthropochory) into the hollow. Remains carried by melt water to the site, could only have arrived from the hydrological catchment of the site. The catchment is small (about 3000m^2^), due to the location just below the main Alpine ridge. Remains of plants growing in this area can be considered of local origin. Other remains are blown into the site by wind or are transported by human or animal activity. Their provenances might exceed the local catchment and may even derive from the valley bottoms. Such remains are defined as extra-local. The representativeness of a bryophyte fragment recovered from the hollow depends on abundance in the vegetation, distance from the growing area and its preservability over thousands of years after deposition.

### Radiocarbon dating

Eleven of the radiocarbon dates relevant to the Iceman pertain to the mosses. They fall between about 4, 000 and 2,500 BC. Details are in S2 Appendix.

### Excavations and sampling

The samples with bryophytes from the Iceman site were recovered in the course of two archaeological excavations at the discovery site conducted by Andreas Lippert, University of Vienna. The first excavation took place immediately after the discovery and lasted only three days due to bad weather conditions in autumn 1991. It was restricted to the south-eastern part of the gully, where the body had lain [32, 33]. The second excavation took place in the summer of 1992and focused on the bottom ice layer incorporating the most of the finds [34]. During this campaign the complete southern part of the gully was unearthed. The sun mainly melted the ice; only if artefacts appeared in the ice was a steam jet used to thaw out the objects. All melt-water from the gully was collected and directed with a system of gutters into a set of sieves, which collected all small finds carried with water. At the beginning of the excavation, a 1 m grid covering the gully was surveyed from the surface and coordinates were measured for detailed documentation and precise spatial information to ensure the exact location of all material retrieved from the site. After all the ice had been removed from the gully, the sediment of each quadrant was also sampled and taken to the Institute of Botany in Innsbruck for further analysis.

All discoveries were listed in a catalogue and ordered by the following categories of finding types [34]: 1. Equipment: comprising the wooden tools and weapons, such as the bow and the axe, quiver, backpack, birch bark vessels containing charcoal and maple leaves, retouching tool, wood; 2. Clothing: including twisted and sewn leather, fur, hat, shoes; 3. Netting: grass cape, twisted grasses, cords, animal hair, grass leaves, 4.Stray finds: summarize all finds, that could not be assigned to any of the categories mentioned;. 5. Sediment: all remains—mainly minerogenic but also organic—retrieved from the bottom of the gully. We use this classification also in our multivariate analyses. For further details on the samples (position at the discovery site, designation, finding type, etc.) see S1 Table.

### Field survey of bryophytes

With emphasis on those species recovered with the Iceman, the present day bryoflora has been extensively recorded in one km squares in the region as shown in a few distribution maps already published [18, 22].These maps are now greatly augmented by the number of recorded squares much increased to 200.The bryophytes growing in the nival zone, above 3000m a.s.l, are listed in S3 Appendix.

### Multivariate analyses

Two multivariate statistical approaches using the computer program CANOCO 4.5 for Windows were used for the analysis of the data set: Principal Component Analysis (PCA) and Redundancy Analysis (RDA), both with linear response models [35]. Two exploratory Detrended Correspondence Analyses (DCA) using the data sets for the PCA and RDA show gradient lengths between 2–3 SD (standard deviation units = taxonomic turnover units) indicating that the linear models PCA and RDA were appropriate for the bryophyte data-set [36].

The PCA Ordination is based on square root transformed count data including 200 samples with 70 different bryophyte species from the Iceman discovery site at 3210 m above sea level and 1 sample including 4 different mosses from the Iceman´s intestines. The two variables “moss stems” and “Hepaticae” were included in the analysis as supplementary parameters. The reduction of the complex multivariate dataset to 2 Principal Components (PC) highlights the relationship among the bryophyte species.

For the bryophyte distribution in relation to explanatory parameters a Redundancy Analysis (RDA) was performed [35] to find out the power of the explanatory parameters. The bryophyte data-set for the Redundancy Analysis comprises the same 200 samples and the analysis is based on square root transformed raw count data. Apart from the 70 bryophyte species also 14 types of macrofossils showing in part high frequency (animal hair, *Brachypodium* spikelets, cord, *Corylus* wood, *Picea/Larix* wood, Poaceae/Cyperaceae/*Juncus* leaves, leather twisted, *Tilia* bast, *Tilia* wood, charchoal sum, crops, aves (bird) feathers, faeces, *Acer platanoides* leaves) were included in the analysis. The following explanatory variables following 0/1 coded parameters were used: sediment, clothing, netting, equipment and stray finds. These parameters indicate the site of retrieval or collection of the samples. Since collinearity among the parameters poses a problem for multivariate analysis an examination of the correlation matrix between these variables was carried out to detect redundant variables. Apart from the low correlations between the variables sediment, clothing, netting, equipment and stray finds, they are also characterised by low variance inflation factor values (VIF < 20) implying that they were less redundant among each other [35]. The statistical significance of the canonical axes and the explanatory variables were determined using Monte Carlo permutation tests with 499 random permutations [35, 37].

Apart from the Iceman intestine samples (red square) the sample numbers are not shown in both figures of the PCA and RDA to avoid overcrowding of the graphs. In the RDA ordination the direction and the length of the explanatory variables (bold red arrows) indicate the importance and the correlation of the variables to each other and to the first two RDA axes. The position (angle) of the species scores (blue arrows) to the explanatory variables (bold red arrows) shows the relationship of the species to the explanatory variables.

## Results

Based on the assumptions above, we tested the hypothesis that abundantly recorded bryophyte taxa have to be of local origin. Therefore we used a PCA to segregate the local from the extra-local bryophyte taxa. The discriminating criterion is the frequency of a taxon in the samples. We used all samples with their species and their counts. To avoid an over- or under-representation of any species we used a square root transformation of the counts. This analysis results in a segregation of local and extra-local species, whereby the first three axes of the PCA accounted 80.8% of the variance in the species data (axis 1: 70.1%, axis 2: 6.4% and axis 3: 4.3%).

**Fig 5:**
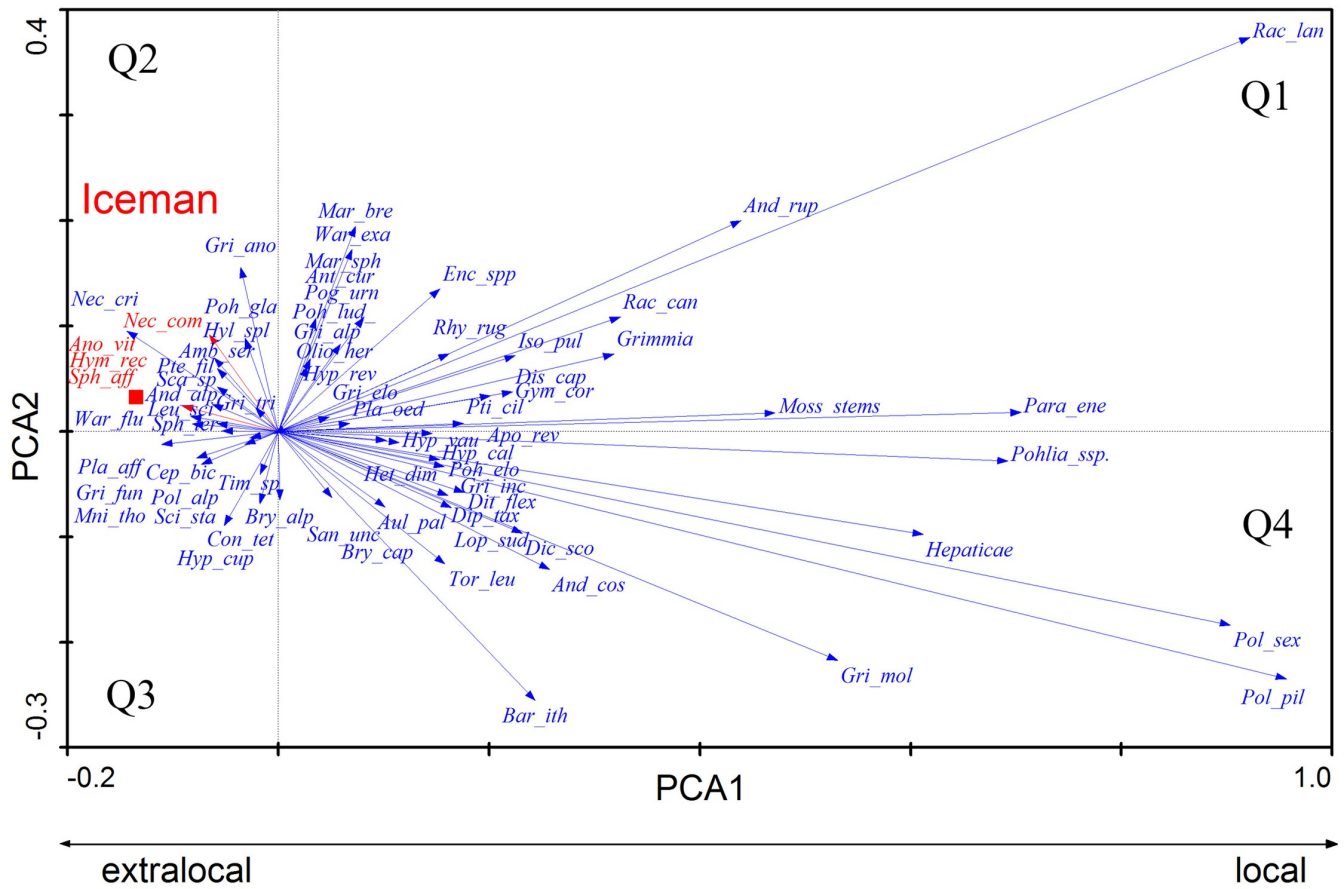
Principal Component Analysis (PCA) of all bryophytes retrieved from 200 samples of the recovery site: Bryophytes occurring on the site (local) are grouped at the right side, whereas those transported to the site (extralocal) are disposed left. Only the Iceman´s intestine samples (red square) are displayed, all other samples are left out to avoid overcrowding. The first three axes of the PCA accounted 80.8% of the variance in the species data (axis1: 70.1%, axis2: 6.4% and axis3: 4.3%). The few names in red are those mosses recovered from the Iceman’s alimentary tract. The formal bryophyte names (in blue) are abbreviated and mean: Amb_ser = *Amblystegium serpens*, And_alp = *Andreaea alpestris*, And_cos = *Andreaea costae*, And_rup = *Andreaea rupestris*, Ano_vit = *Anomodon viticulosus*, Ant_cur = *Antitrichia curtipendula*, Apo_rev = *Apomarsupella revoluta*, Aul_pal = *Aulacomnium palustre*, Bar_ith = *Bartramia ithyphylla*, Bry_alp = *Bryum alpinum*, Bry_cap = *Bryum capillare*, Cep_bic = *Cephalozia bicuspidata*, Con_tet = *Conostomum tetragonum*, Dic_sco = *Dicranum scoparium*, Dip_tax = *Diplophyllum taxifolium*, Dis_cap = *Distichium capillaceum*, Dit_flex = *Ditrichum flexicaule*, Enc_spp = *Encalypta* indet, Gri_alp = *Grimmia alpestris*, Gri_ano = *Grimmia anodon*, Gri_elo = *Grimmia elongata*, Gri_fun = *Grimmia funalis*, Gri_inc = *Grimmia incurva*, Gri_mol = *Grimmia mollis*, Gri_tri = *Grimmia triformis*, Grimmia = *Grimmia indet* Gym_cor = *Gymnomitrion corallioides*, Heps = liverworts, Het_dim = *Heterocladium dimorphum*, Hyl_spl = *Hylocomium splendens*, Hym_rec = *Hymenostylium recurvirostrum*, Hyp_cal = *Hypnum callichroum*, Hyp_cup = *Hypnum cupressiforme*, Hyp_rev = *Hypnum revolutum*, Hyp_vau = *Hypnum vaucheri*, Iso_pul = *Isopterygiopsis pulchella*, Leu_sci = *Leucodon sciuroides*, Lop_sud = *Lophozia sudetica*, Mar_bre = *Marsupella brevissima*, Mar_sph = *Marsupella sphacelata*, Mni_tho = *Mnium thomsonii*, Moss_st = moss stems, Nec_com = *Neckera complanata*, Nec_cri = *Neckera crispa*, Olio_her = *Oligotrichum hercynicum*, Para_ene = *Paraleucobryum enerve*, Pla_aff = *Plagiomnium affine*, Pla_oed = *Plagiopus oederianus*, Pog_urn = *Pogonatum urnigerum*, Poh_elo = *Pohlia elongata*, Poh_gla = *Pohlia wahlenbergii var*. *glacialis*, Poh_lud_ = *Pohlia ludwigii*, Pohlia_spp = *Pohlia* indet, Pol_alp = *Polytrichum alpinum*, Pol_pil = *Polytrichum piliferum*, Pol_sex = *Polytrichum sexangulare*, Pte_fil = *Pterigynandrum filiforme*, Pti_cil = *Ptilidium ciliare*, Rac_can = *Racomitrium canescens s*.*l*., Rac_lan = *Racomitrium lanuginosum*, Rhy_rug = *Rhytidium rugosum*, San_unc = *Sanionia uncinata*, Sar exa = *Sarmentypnum exannulatum*, Sca_sp = *Scapania* indet, Sci_sta = *Sciurohypnum starkei* (*Brachythecium starkei*), Sph_aff = *Sphagnum affine*, Sph_ter = *Sphagnum teres*, Tim_sp = *Timmia* indet, Tor_leu = *Tortula leucostoma*, War_flu = *Warnstorfia fluitans*.

On the right side in the quadrants Q 1 and Q4 all abundant species are grouped (Fig 5 PCA local/extra-local; the number of samples, in which a species is reported, is given in Supplementary Information 2). This comprises mosses retrieved copiously (hundreds to thousands pieces) with high frequency (>40% of all samples): *Pohlia* spp., *Polytrichum piliferum*, *Polytrichastrum sexangulare* and *Racomitrium lanuginosum*. These five all occur in the catchment of the discovery site and can grow at even greater altitudes up towards the peaks close to 3500 m. Furthermore, those species, which were retrieved from 5% or more of the samples, grow in the nival zone of Alps today and most were recorded at or near the site (see S3 Appendix). Many species found only as single fragments are also fully capable of inhabiting the nival zone.

**Fig 6:**
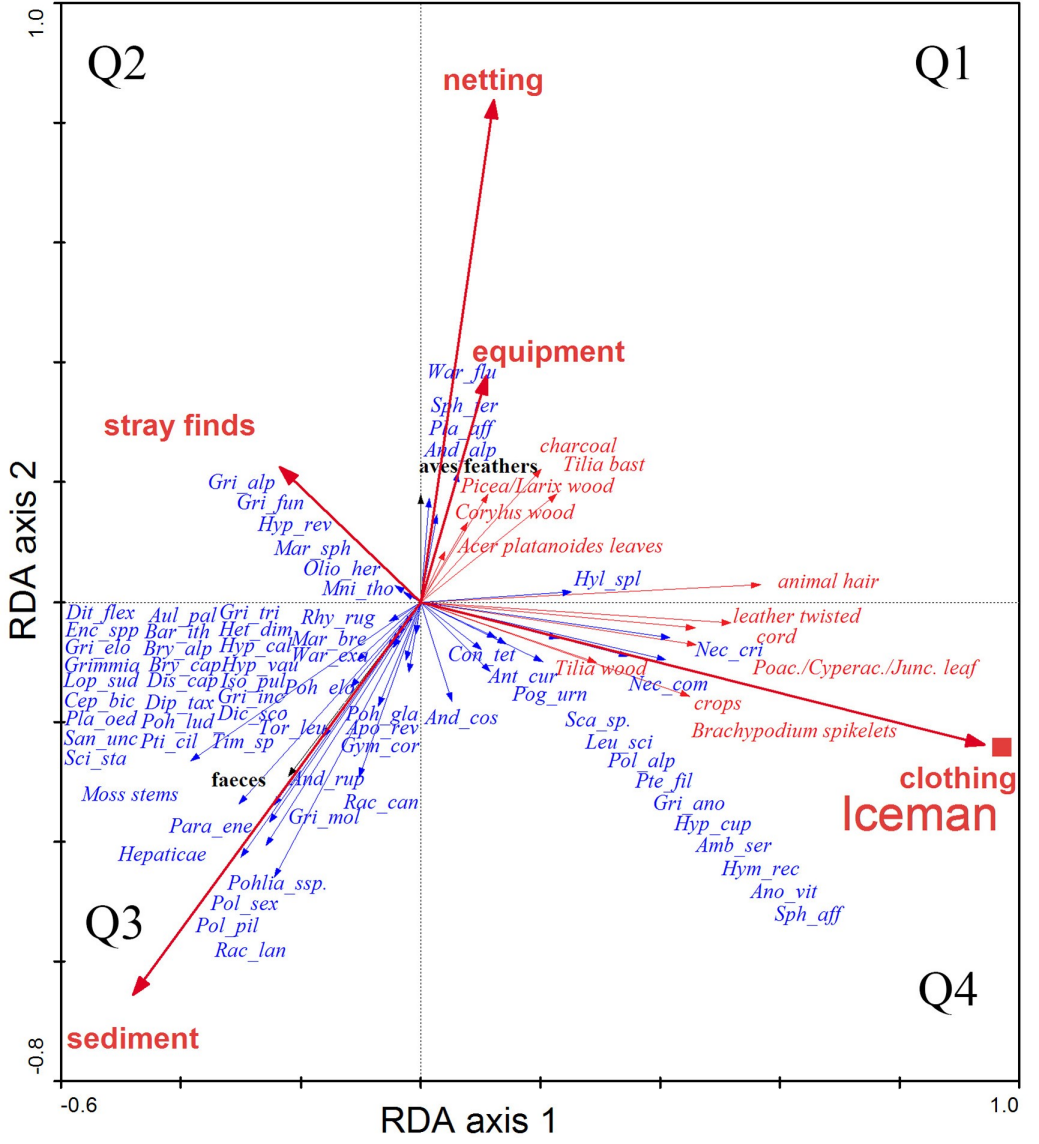
Redundancy analysis (RDA) of all bryophyte samples from the discovery site. Arrows indicate explanatory variables, red ones refer to items of the Iceman except for sediment deposited at the site; black ones relate to items transported by animals to the site. For abbreviations of the formal bryophyte (blue arrows) names see Fig 5.

**Fig 7:**
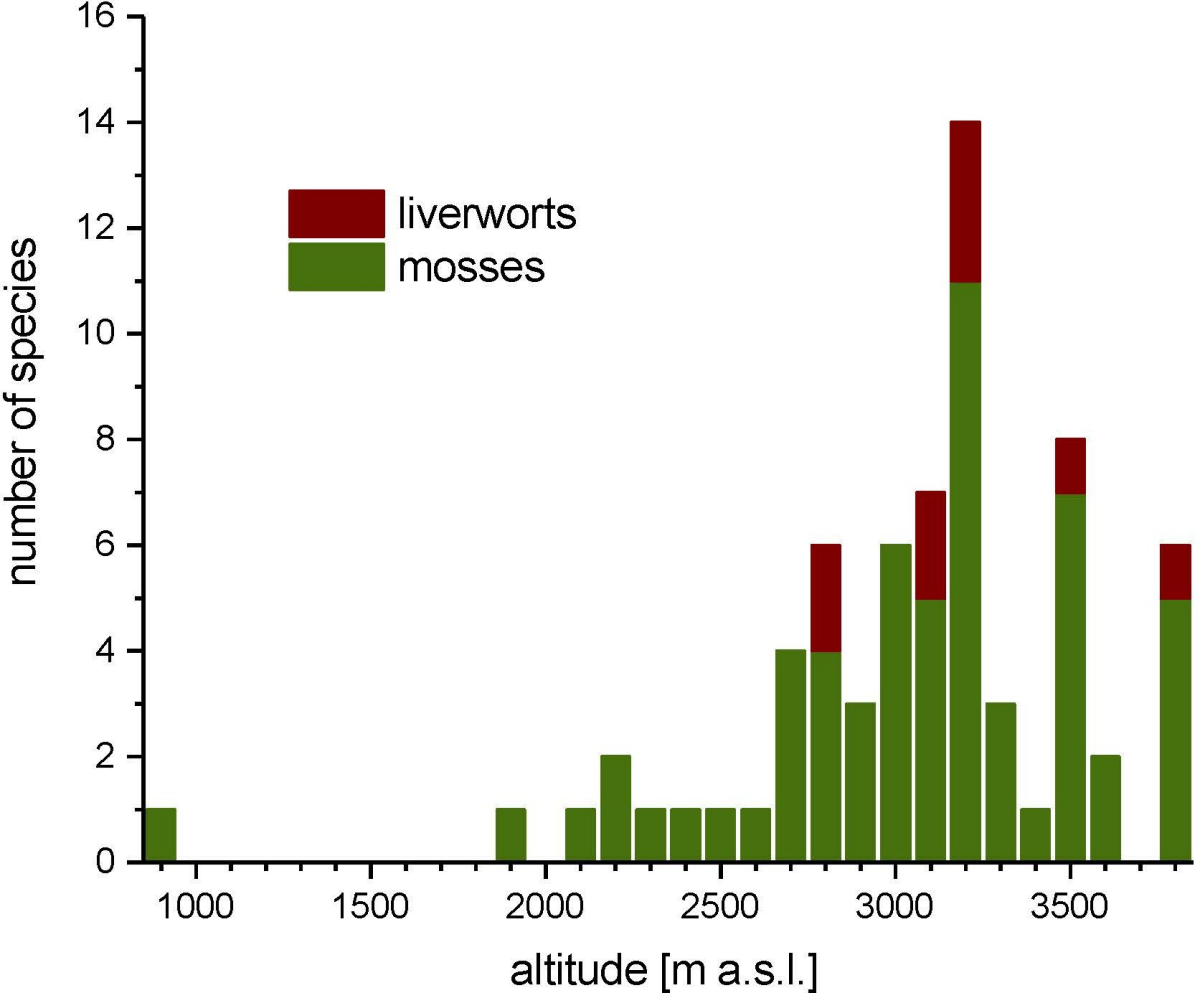
Altitudinal limits. Using data from Tyrol and Austria as a whole, maximal altitudinal distributions at present of the subfossil bryophytes recovered from the Ötzi site at 3,200m above sea level. 68% of the species grow in the nival zone while 32% have not been recorded in that zone.

A special case is *Neckera complanata* (Q2, Fig 5) which is recorded in almost 5% of the samples. This species has a present day colline/montane distribution (reaching 1,750m in Schnalstal and only 1,900m in all Austria) and was transported to the site by the Iceman. It grows nowhere near the nival zone (Figs 5 and 6). Slightly more than two thirds of the species have been reported from the nival zone while slightly less than one third have not (Fig 7).

However, 51 bryophytes occurred only in single or few samples and have a frequency below 5%.The PCA provides no distinct information if these bryophytes are local or extra-local. Therefore we correlated the mosses with the category of finding types listed above (See Methods) to reveal the processes which transported the bryophytes to the site. For this purpose we used an RDA (Fig 6). Again, the analysis was conducted with the square roots of the counts, that high numbers are not overestimated. The RDA based on the first two axis explains 47.5% (axis 1: 33%; axis 2: 14.4%) of the variance in the species data and 74.2% in the fitted species data. For the species analysis along with the explanatory variables the unrestricted Monte Carlo permutation tests (499 permutations) of the significance of the canonical axes indicate axis 1 as statistically highly significant (p = 0.002). The significance value of all canonical axes indicates the relation between species and explanatory variables as highly significant (p < 0.002). According to the unrestricted permutation tests with forward selection the two parameters clothing (p = 0.002) and netting (p = 0.016) are statistically significant and are the main explanatory variables for the distribution of the bryophytes and macrofossils of the studied site. The ordination is done with the remains recovered first, then the bryophytes and all other categories were added.

This results in two major groups: Group 1. On the right side in Q1 and Q4 all bryophytes grouping with the Iceman are shown. There exists a high probability that these bryophytes came to the discovery site with the Iceman, because the mosses correlate significantly with his gear. The asterisks * in the following paragraphs indicate those species that could not have grown in the nival zone. In Q1 Warnstorfia fluitans*, Sphagnum teres*, Plagiomnium affine* and *Andreaea alpestre* correlate with netting and equipment, indicating that they adhered to items of these two categories. *Sphagnum teres* derives from one sample (grasses, hair, cords) and *Warnstorfia fluitans* comes also from one sample (cords, hair, fibres) with both attributed to the netting category. *Plagiomnium affine* and *Andreaea alpestre* were discovered in the context of the birch bark vessel (two samples) indicate that they had adhered to the equipment. However, feathers are also recorded in the samples with these mosses. This indicates a minor correlation with feathers suggesting the slight possibility that they have been transported by birds to the site.

The mosses in Q4 show a strong relation to clothing: Neckera crispa* is found in 6 samples (5 designated as leather, cords) which refer to clothing and one sample (designated as cords, hair, fibres) belonging to the backpack. Neckera complanata* occurs in 9 samples, four referring to clothing, three to sediment and two to stray finds; additionally it occurs in the intestines samples. Furthermore, this quadrant includes *Conostomum tetragonum*, which occurred in three samples on the clothing of the Iceman and in the sediment, Antitrichia curtipendula* two samples of his clothing and in two sediment samples. *Pogonatum urnigerum* is recovered from 14 samples allocated to clothing in three samples, in 10 sediment samples and to netting in one sample.

Furthermore Leucodon sciuroides*, *Polytrichastrum alpinum*, *Pterygynandrum filiforme*, *Grimmia anodon*, Hypnum cupressiforme* and Amblystegium serpens* are retrieved from clothing, although some of these mosses occur also in sediment and other samples. *Andreaea (*costate spp probably *nivalis*) occurs in 48 samples: 34 from sediment, 5 from stray finds, 5 from clothing, 3 from netting and one from equipment. This distribution in the different samples is due to the fact that some moss fragments might have been dislocated, when the ice in the gully was melted [14].

Hymenostylium recurvirostre*, Anomodon viticulosus*, *Sphagnum cf*. affine* were all recorded only in his intestine samples.

Of these mosses *Amblystegium serpens*, *Anomodon viticulosus*, *Antitrichia curtipendula*, *Hymenostylium recurvirostre*, *Hypnum cupressiforme*, *Leucodon sciuroides*, *Neckera complanata*, *Neckera crispa*, *Pterygynandrum filiforme* and *Sphagnum affine* have altitudinal limits of well below 3,000m and could not have grown at the site. However, these low to moderate altitude bryophytes potentially tell us a lot about the habitats the Iceman crossed when he ascended to the Tisenjoch.

Group 2. The mosses on the left side (Q2 and Q3) arrived at the site in a different way. The upper quadrant Q2 displays mosses which correlate mainly with stray finds. *Grimmia alpestris* (one sample, hair, bast, mosses), *Grimmia funalis* (one sample, hairs), *Hypnum revolutum* (one sample, hair, bast, mosses), *Marsupella sphacelata* (two samples, melt water; one sample sediment; one sample, grasses, bast, faeces), *Oligotrichum hercynicum* (one sample hair, bast, mosses), *Mnium thomsonii* (one sample of hairs; this is the only sample of the stray finds, which contains a feather). This is poor evidence that mosses were transported to the site by birds.

The lower quadrant Q3 shows bryophytes which correlate with the sediment and faeces. These alpine and nival mosses have a high probability of having been brought to the site by wild game such as alpine ibex.

The pluses + in the following list indicate those species which do grow at the site or nearby now (above 3,000m). Of these bryophytes *Andreaea rupestris+*, *Apomarsupella revoluta*, *Bartramia ithyphylla+*, *Bryum cf*. *alpinum*, *Cephalozia bicuspidata+*, *Dicranum cf*. *scoparium*, *Diplophyllum taxifolium*, *Distichium capillaceum*, *Ditrichum flexicaule*, *Grimmia elongata+*, *Grimmia incurva+*, *Grimmia mollis*, *Grimmia triformis+*, *Gymnomitrion corallioides+*, *Hypnum callichroum*, *Hypnum vaucheri+*, *Lophozia sudetica+*, *Marsupella brevissima+*, *Paraleucobryum enerve+*, *Rhytidium rugosum*, *Pohlia elongata*, *Pohlia wahlenbergii var*. *glacialis+*, *Pohlia ludwigii*, *Polytrichum piliferum+*, *Polytrichastrum sexangulare+*, *Racomitrium canescens s*.*l*.*+*, *Racomitirum lanuginosum+*, *Sanionia uncinata* and *Tortula leucostoma* have the potential to grow today at the site.

*Aulacomnium palustre*, *Bryum capillare*, *Heterocladium dimorphum*, *Isopterygiopsis pulchella*, *Plagiopus oederianus*, *Ptilidium ciliare*, *Sciuro-hypnum starkei* and *Warnstorfia exannulata* have their altitudinal limits distinctly below 3,000m and grow mainly in the alpine and subalpine belts or lower. Again it is possible that these too have been transported to the site by wild game such as alpine ibex.

## Discussion

### Differential preservation

The Iceman site is unique in Quaternary and archaeological bryology, whither in North America or Europe where such work has been carried out for many decades since the 19^th^ century [38, 39, 40]. No other site has been investigated at such a great altitude with bryophytes preserved by freezing over thousands of years. The rich bryophyte assemblage is especially outstanding in the large number of liverworts and the suite of rock-loving species such as *Andreaea* spp. and particularly *Grimmia* spp. Seven species of the latter genus is an unparalleled discovery from a Quaternary site.

The under-representation of liverworts in Quaternary and archaeological assemblages has been a recurrent topic and the Iceman bryophytes bear greatly on this matter. Subfossil bryophyte assemblages totalling many species often include not a single liverwort. In this case the implication is that rapid freezing has allowed the preservation of more than ten species of liverworts. The presence of frozen fragments of liverworts in samples taken from two rock glaciers, one in Switzerland and one in South Tyrol, supports the freezing theory for good representation of liverworts, better revealing the former abundance in the vegetation [41, 42].

### Anthropochory

**Fig 8:**
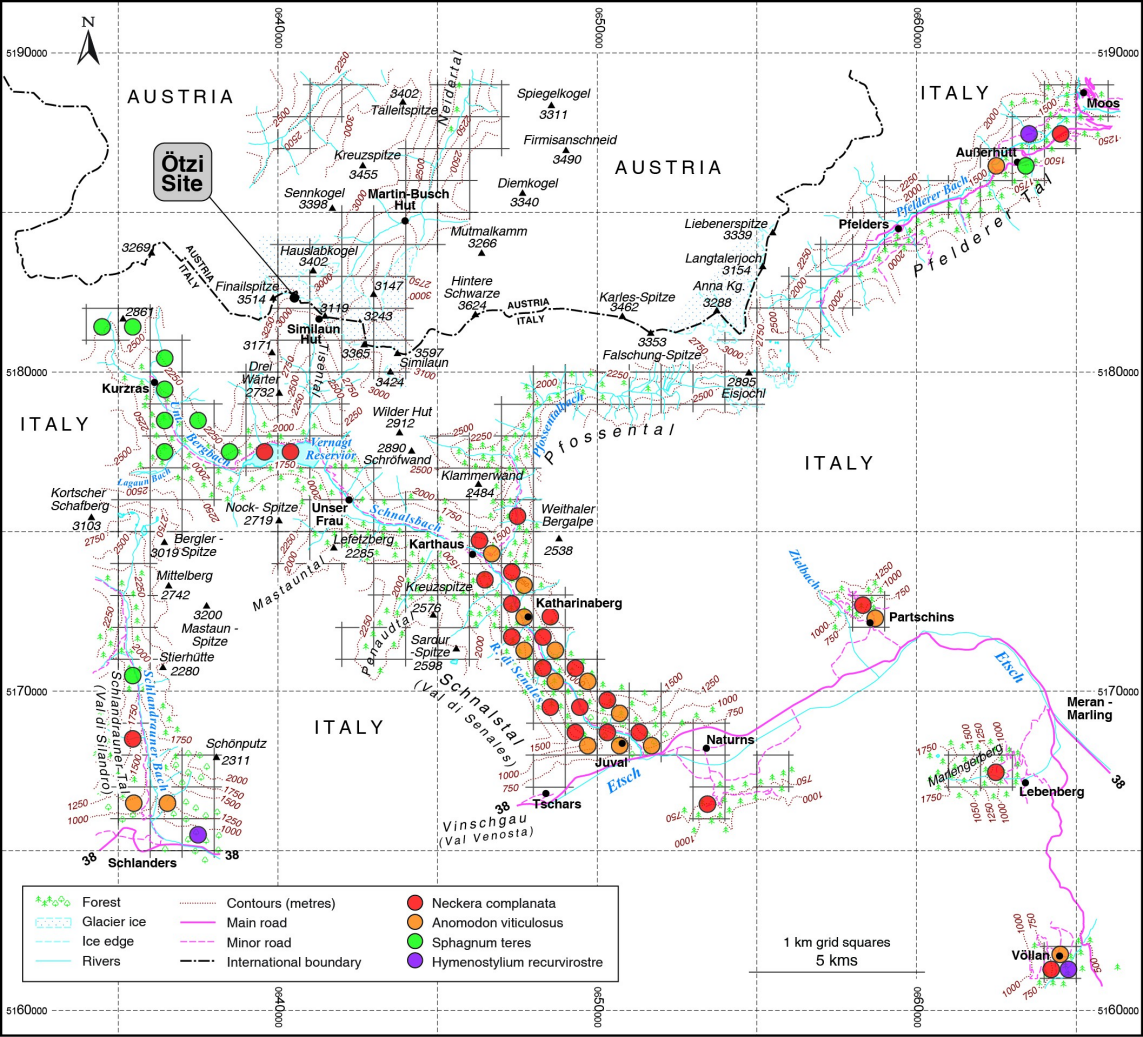
Moss distribution. Map showing the present distribution of the four mosses *Anomodon viticulosus*, *Hymenostylium recurvirostre*, *Neckera complanata* and *Sphagnum teres* in the region of the Iceman site.

Endoanthropochory is indisputable for a small number of mosses. That the Iceman carried mosses unintentionally to the discovery site is certain because there are very small fragmentary remains of several mosses in his alimentary tract, most notably *Neckera complanata*, *Anomodon viticulosus*, *Hymenostylium recurvirostre* and two species of bogmosses, *Sphagnum* sp. and *Sphagnum affine* [16, 21, 22]. See S4 Appendix. The most intriguing among these species is *Sphagnum affine* which occurred in only one of the colon samples. *Hymenostylium recurvirostre* and *Sphagnum affine* are unexpected discoveries, discussed at length by Dickson et al. [21, 22]. *Sphagnum affine* is unknown now in South Tyrol but could well have grown in Vinschgau where formerly there were extensive mires. Had he known its absorbent, antiseptic properties, the Iceman could have gathered it there at 800m above sea level. Often lime-encrusted, *Hymenostylium recurvirostre*, found growing in only three squares (Fig 8), occurs in abundance only on wet, basic rock. How it came to be in the Iceman’s lowermost gut is a difficult matter to explain; perhaps in drinking water?

The Iceman is not alone in having moss fragments internally. The alimentary tracts of the two most famous Danish Iron Age bog bodies both have *Sphagnum* fragments as does Lindow Man from England [5]. Long Ago Person Found, a glacier mummy from British Columbia, had *Sphagnum* cf *Acutifolia* as well as *Andreaea* and several other mosses in the innards [5, 43]. In none of these cases were the mosses ingested deliberately. Neither nutritious nor palatable mosses are not knowingly ingested and certainly not as staples [44]. With bogmoss leaves and a few other mosses in her faeces, perhaps an exception is the late 15^th^ century Greenlandic frozen mummy of a woman who may have been starving to death which the Iceman certainly was not [5].

Before the moss fragments were known from the Iceman’s alimentary tract [16], that he had deliberately carried *Neckera complanata* was beyond reasonable doubt from the large mass of that species from the sample 91/124 (“sewn leather, fur, grasses”, belonging to his clothing; [17]). Another example is 91/137 leather, grasses, hairs from clothing had *Neckera complanata*, *Neckera crispa*, *Hylocomium splendens Pohlia sp* and *Andreaea* probably *nivalis*. This is the earliest known example of both ectoanthropochory and endoanthropochory.

### Zoochory

That large mammals such as alpine ibex or chamois traversing or lingering at the Iceman site brought bryophyte fragments on feet or hair is surely very likely. That was ectozoochory and, furthermore, endozoochory is proven by the discovery of moss fragments in caprine droppings from the site. One dropping contained a fragment of *Heterocladium dimorphum* and another contained a fragment of *Polytrichum piliferum*. See S5 Appendix. *Heterocladium dimorphum* is not known to grow now in the Austrian Alps at higher than 2,800m [45] being a height 200m short of the nival zone and some 400m short of the Iceman site. Because of its habitat often being crevices in rock, it is not a particularly expected moss to have been inside a large grazer. In contrast *Polytrichum piliferum* is no surprise, this being a very common moss over a great altitudinal range and very frequent in the nival zone. Remains of mosses have been recovered from archaeological caprine droppings elsewhere, but only very sparsely, for example Akeret and Jacomet [46] and Jacomet, Leuzinger and Schibler [47]. Goats and sheep do not deliberately eat bryophytes and therefore few in quantity or variety are to be expected. Even larger herbivores such as muskoxen and reindeer/caribou eschew mosses if the preferred lichens are available [48].

### Hydrochory

This was important only for local species. However, the catchment of the Iceman site is small and movement by water can only have taken place during the summer melt of snow and ice; the thousands of fragments *Polytrichum*. *piliferum*, *Polytrichastrum sexangulare* and *Racomitrium lanuginosum* may well have been carried by meltwater. At present, however, there are no constantly fast-flowing streams in the catchment. In the late summer meltwater merely seeps into the hollow.

Anemochory. Among the many wind-blown mosses listed in the papers by Miller and Howe Ambrose [29, 30] and McDaniel and Miller [31] are the nival or alpine zone mosses found with the Iceman such as *Polytrichum piliferum*, *Hypnum revolutum*, *Hypnum vaucheri* and *Ditrichum flexicaule*. Wind could have blown fragments of local bryophytes to lodge in the snow in or on top of the snow-filled hollow not just in summer but at other seasons. Flø and Hägvar found moss fragments including bulbils to be “common” in sticky traps very close to a glacier in southern Norway. Such aerial transport was mainly below 0.5m [49].

There is little evidence that bryophyte leafy fragments are transported over very large distances as distinct from microscopic spores which certainly are but of no relevance here. Even if a fragment of any of the 75 species had arrived from very far off there is no way that that could be known.

### Last journey

An ascent along the lower Schnalstal was suggested by Lippert [32] and is in congruence with the reconstruction of his last itinerary by pollen and moss fragments from his intestines [5, 15]. The small tree Hophornbeam (*Ostrya carpinifolia*), the pollen of which was identified in big quantities in the large bowel, occurs in the lower Schnalstal in the forests. It seems puzzling that he took the most stressful track through a gorge, but considering scenarios that he was on the run [2, 50] a gorge provided most opportunities to hide. There are five mosses of especial importance in this matter (Fig 8): *Neckera complanta*, *Anomodon viticulosus*, *Hymenostylium recurvirostre*, *Sphagnum teres* and *Sphagnum affine*.

To the north of the Iceman site, *Neckera complanata* has not been recorded in Ventertal nor even in Ötztal; it is known in the very distant Inntal, about 50 km north of the site as the crow flies and longer on the ground. These five are low to moderate altitude mosses. Mainly of woodlands, the *Neckera* and the *Anomodon* often grow together in the bryosociologial category Neckerion complanatae, as do other Iceman mosses *Neckera crispa*, *Anomodon curtipendula*, *Leucodon sciuroides* and *Amalystegium serpens*. Today these mosses thrive in the lower Schlandraunertal, the lower Schnalstal and in the surroundings of the Vernagt reservoir (Fig 8). They are most frequent from the gorge of the Schnalstal to the mouth of the Pfossental. This strengthens the claim that the Iceman went up Schnalstal on his very last ascent, though no *Hymenostylium* has been found there: that calcicole moss of wet rock is known very near Schlandraunertal and in Pfelderertal too (Fig 8). A journey from the distant Pfelderertal seems very unlikely but the possibility of Schlandraunertal cannot be totally excluded.

Like most species of *Sphagnum*, *Sphagnum teres* grows in mires. There is an area with this species in several contiguous squares at the northwest (Kurzras and nearby, Fig 8). The aquatic/mire species *Warnstorfia exannulatus* (*Sarmenthypnum exannulatum*) and *W*. *fluitans* both grow near Kurzras. Perhaps this indicates that the Iceman was in that vicinity just before the ascent to 3,210m and his violent death. For this very last climb either Finailtal or Tisental would have been possible. Before all that, the fragment of the low altitude, mire species *Sphgnum affine* taken from the colon is an indication that Ötzi had been in the Vinschgau valley bottom, conceivably as low as 600m; that after his first descent and just before the final ascent to the nival zone [2,5,15].

### Palaeoecology

In the bryophyte assemblage there are rock-loving species, aquatic species, mire species, bare ground species and shade-loving woodland species. The local species are mainly acidophytes and cryophytes, both chionophile and chionophobe [51].

The subfossil bryophyte record from the hollow suggests that the bryoflora between 4,000 and 2,000 years BC was very similar to that growing now (S1 Appendix and S1 Table). With over 3,000 recovered fragments wide ranging *Polytrichum piliferum* in 182 samples out of 200 was abundant then as now, the same for the chionophilous *Polytrichastrum sexangulare* in 137 samples. This correspondence can be claimed also for the chionophobous *Paraleucobrym enerve* in 74 samples. As now there was a boulder strewn landscape with much bare rock and summer snow melt but plenty snow lying late into summer and through the year.

However, there are some marked contrasts. Forming merely small tufts here and there, this present scarcity of *Racomitrium lanuginosum* in the vegetation contrasts with the thousands of fragments from 148 samples. Even more of a contrast is the good subfossil representation of *Grimmia mollis* in 53 samples with a total lack from the hydrological catchment now; it is a species of more or less constantly wet rock and sides of rivulets. That type of habitat similarly suits *Andreaea nivalis* in 48 samples while being very sparse in the catchment now. It also suits *Apomaruspella revoluta* in 11samples; that liverwort is not in the catchment now. However, it is known in the nival zone or very close to it elsewhere in Austria and Italy, Switzerland and France. All that suggests that the catchment was wetter for much or all of the time covered by the subfossils. If that is a correct deduction then the case for hydrochory is supported.

## Conclusions

The discovery of subfossil bryophytes recovered from the Iceman site is without parallel in Quaternary bryology. Thousands of fragments representing at least 75 mosses and liverworts became frozen in the rocky hollow between about 4,000 and 2,000 BC, a 2,000 year period in which the climate fluctuated but perhaps not enough to have markedly changed the bryoflora.

The subfossil bryophytes provide cogent evidence that the Iceman’s last journey was northwards up Schnalstal to the vicinity of Vernagt before the final climb to 3,210m above sea level near Hauslabjoch.

About 30% of the subfossil species were local, nival zone bryophytes (from within the small hydrological catchment), the rest extra-local. About 70% were brought to the site by the Iceman and large herbivores traversing the colline, montane, alpine and nival zones.

## Supporting information

S1 Appendix List of Bryophytes preserved by Freezing. (PDF)

S2 Appendix Radiocarbon Dates of Mosses. (PDF)

S3 Appendix List of Bryophytes growing in the nival zone now. (PDF)

S4 Appendix Mosses recovered from the Iceman’s Intestines. (PDF)

S5 Appendix Mosses recovered from Caprine Faeces. (PDF)

S1 Table Bryophyte samples analysed from the discovery site. Besides the bryophyte species and their counts the x/y-coordinates, altitude, quadrants, designation of the findings and finding-type are given according to Bagolini et al. [34].
(XLS)
