# Supplementary material for: Seventy-five mosses and liverworts found frozen with the late Neolithic Tyrolean Iceman: Origins, taphonomy and the Iceman’s last journey
Source: PLoS One. 2019 Oct 30;14(10):e0223752. doi: 10.1371/journal.pone.0223752 (PMC6821077; doi:10.1371/journal.pone.0223752)
Supplement: S1 Appendix — (PDF) [file pone.0223752.s001.pdf]

S1 Appendix. List of Bryophytes preserved by Freezing

List of all Bryophytes recovered frozen from periods before, during and after the time of the Iceman. Nomenclature follows [52] and [53].

The taxa are listed in decreasing order of abundance showing total number of fragments, presence in samples out of 200 and length of longest stem in mm. So the most abundant moss *Polytrichum piliferum* totalled 3,181 fragments, was retrieved from 182 samples out of 200 and the longest fragment measured 12mm.

Polytrichum piliferum 3,181;182;12. *Racomitrium lanuginosum* 2,377;148;10. *Polytrichastrum sexangulare* 1,240;137;8. *Pohlia* spp 599;138;mostly a few mm but to 20mm. *Paraleucobryum enerve* 144;74;8. *Grimmia mollis* 92;53;2. *Andreaea* ecostate (mostly *rupestris*) 262;77;8. *Andreaea costate* (mostly or entirely *nivalis*) 88;48;5. *Racomitrium canescens* s.l. 30; 20; 2. *Grimmia* spp 47; 35; mostly a few mm. *Dicranum scoparium* 19;17;5. *Pogonatum urnigerum* 15; 14;2. *Grimmia incurva* 12; 12; 10. *Distichium capillaceum* 17;13; 2. *Hylocomium splendens* 20;12;4. *A. revoluta* 12;11;4. *Gymnomitrion corallioides* 19;10;4. *Neckera complanata* 83;10;60. *Polytrichastrum alpinum* 8;8;6. *Rhytidium rugosum* 7;7;2. *Neckera crispa* 8;6; 22. *Hypnum revolutum* 5; 5; 8. *Ditrichum flexicaule* 4; 4;10. *Lophozia sudetica* 4;4;4. *Marsupella sphacelata* 4; 4;3. *Encalypta* sp or spp 3; 3; 13. *Antitrichia curtipendula* 3;3;4. *Conostomum tetragonum* 3;3;3. *Gimmia elongata* 3;3; 6. *Aulacomnium palustre* 2; 2; 2. *B.*

ithyphylla 2; 2;7. *Grimmia anodon* 2; 2;3. *Plagiomnium affine* 2;2;6. *Pohlia elongata* 2;2;22. *Pohlia wahlenbergii glacialis* 2;2;4. *Ptilidium ciliare* 2;2;5.

All the following species were found as only one fragment. The length of the fragment is given in mm.

Amblystegium serpens 6. *Andreaea alpestris* 2. *Bryum* sp 5. *Bryum cf. alpinum* 7. *Bryum* cf *capillare* 3. *Cephalozia bicuspidata* 2. *Diplophyllum taxifolium* 4. *G. alpestris* 3. *G. funalis* 9. *Grimmia triformis* 3. *Heterocladium dimorphum* 2. *Hymenostylium recurvirostrum* 2. *Hypnum callichroum* 2. *H. cupressiforme* 2. *Hypnum vaucheri* 2. *Isopterygiopsis pulchella* 6. *L. sciuroides* 4. *Marsupella* cf *brevissima* 2. *Mmnium thomsonii* 6. *Oligotrium hercynicum* 2. *Plagiopus oederianus* 2. *Pohlia ludwigii* 1 leaf. *Pterygyandrum filiforme* 4. *Sanionia uncinata* 2. *Scapania* sp 3. *Sciuro-hypnum starkei* 1 leaf. *Sphagnum affine* 0.2. *Sphagnum teres* 1.5. *Sphagnum* sp. 0.3. *Timmia* sp 1 leaf. *Tortella* cf. *fragilis* 4. *Tortella* cf. *tortuosa* 8. *Tortula leucostoma* 3. *Warnstorfia exannulatus* (*Sarmenthynum exannulatum*) 7. *Warnstorfia fluitans* 9.

Undoubtedly additional to the list, there are some species represented by poorly preserved, small fragments left unidentified especially liverworts. The total is not less than 75 and may be as many as 80 species.

Henk Greven identified or confirmed the *Grimmia* spp, Mark Hill saw the *Sphagnum affine* and the *Sphagnum cf teres*, Barbara Murray saw some fragments of *Andreaea* and Timo Koponen identified the Mniaceae.

References

52. Grolle R, Long DG. An annotated check-list of the Hepaticae and Anthocerotae of Europe and Macaronesia. J Bryol. 2000;22: 103-140.
53. Hill MO, Bell N, Bruggeman-Nannenga MA, Brugues M, Cano MJ, Enroth J, et al. An annotated checklist of the mosses of Europe and Macaronesia. J Bryol. 2006;28(3): 198-267.
