# Supplementary material for: Seventy-five mosses and liverworts found frozen with the late Neolithic Tyrolean Iceman: Origins, taphonomy and the Iceman’s last journey
Source: PLoS One. 2019 Oct 30;14(10):e0223752. doi: 10.1371/journal.pone.0223752 (PMC6821077; doi:10.1371/journal.pone.0223752)
Supplement: S2 Appendix — (PDF) [file pone.0223752.s002.pdf]

S2 Appendix. Radiocarbon Dates of Mosses

Radiocarbon dated bryophytes from the discovery site of the Neolithic glacier mummy according Kutschera et al. [1]. *Sample number refers to the excavation of Bagolini et al. [34]

^{14}C lab nr	Sample nr*	Material	Species	Location	^{14}C age (yrs BP)	Calibrated time range (yrs BC)
VERA-0627	92/307	bryophyte	*Polytricastrum sexangulare*	embedded in ice of quadrant 42	4970 ± 35	3910–3655
VERA-0055	92/283	bryophyte	*Polytricastrum sexangulare*	sediment of quadrant 43	4700 ± 40	3635–3370
VERA-0608	92/210	bryophyte	*Polytricastrum sexangulare*	sediment of quadrant 65	4510 ± 45	3365–3030
VERA-1695	92/402a	bryophyte	*Polytricastrum sexangulare*	crevices in bedrock of quadrant 3	4420 ± 35	3325–2915
VERA-0622	92/300	bryophyte	*Polytricastrum sexangulare*	sediment of quadrant 61	4360 ± 65	3330–2880

VERA-1694	92/402a	bryophyte	*Polytricastrum sexangulare*	crevices in bedrock of quadrant 3	4325 ± 30	3020–2890
VERA-1698	92/371	bryophyte	*Polytricastrum sexangulare*	square meter south of quadrant 3	4260 ± 45	3015–2690
VERA-0642	92/409	bryophyte	*Polytricastrum sexangulare*	square meter south of quadrant 4	4220 ± 60	2930–2610
VERA-1692	92/409a	bryophyte	*Polytricastrum sexangulare*	quadrant 4	3960 ± 30	2575–2345
VERA-1690	92/402b	bryophyte	*Racomiteiumm lanuginosum*	crevices in bedrock of quadrant 3	3925 ± 30	2490 - 2295
VERA-0623	92/300	bryophyte	diverse species	sediment of quadrant 61	3905 ± 35	2480–2285
