# Supplementary material for: Seventy-five mosses and liverworts found frozen with the late Neolithic Tyrolean Iceman: Origins, taphonomy and the Iceman’s last journey
Source: PLoS One. 2019 Oct 30;14(10):e0223752. doi: 10.1371/journal.pone.0223752 (PMC6821077; doi:10.1371/journal.pone.0223752)
Supplement: S3 Appendix — (PDF) [file pone.0223752.s003.pdf]

S3 Appendix. **List of Bryophytes growing in the nival zone now**

The Bryophytes found growing from 3,019 m at the Similaun Hut along the track to a little beyond the Iceman site at 3,210m and a little higher. The area was visited eight times between 1994 and 2007 by JHD, WKH, RDP, GR, AB, and others.

21 species in the close vicinity of the site*: Andreaea nivalis, Andreaea rupestris, Bryum* sp/spp*, Bryum argenteum, Cephalozia ambigua, Cephaloziella* sp.*, Ceratodon purpureus, Grimmia incurva, Gymnomitrion concinnatum, Gymnomitrium corallioides, Lophozia sudetica, Mielichhoferia elongata, Oligotrichum hercynicum, Paraleucobryum enerve, Pogonatum urnigerum, Pohlia nutans, Pohlia obtusifolia, Polytrichastrum alpinum, Polytricastrum sexangulare, Polytrichum piliferum, Racomitrium lanuginosum.*

16 additional species from between the site and the Hut: *Andreaea alpestris , Andreaea rothii, C. tetragonum, Encalypta microstoma, G. alpestris, Grimmia donniana, Grimmia elongata, G. funalis, Grimmia reflexidens, Grimmia triformis, Hypnum vaucheri, Pohlia drummondii, Syntrichia norvegica, Syntrichia ruralis, Tortula muralis,Tritomaria scitula.*
