# Supplementary material for: Seventy-five mosses and liverworts found frozen with the late Neolithic Tyrolean Iceman: Origins, taphonomy and the Iceman’s last journey
Source: PLoS One. 2019 Oct 30;14(10):e0223752. doi: 10.1371/journal.pone.0223752 (PMC6821077; doi:10.1371/journal.pone.0223752)
Supplement: S4 Appendix — (PDF) [file pone.0223752.s004.pdf]

S4 Appendix. Mosses recovered from the Iceman's Intestines

With preservation ranging from good to very bad, these are all small or tiny leaf fragments recognised on slides prepared for pollen analysis. Colon 1 and 2 corresponds to the transverse colon, Colon 3 derives from the sigmoid colon.

Mosses	Stomach	Ileum	Colon 1	Colon 2	Colon 3	Rectum
Anomodon viticulosus			3			
Hymenostylium recurvirostrum						8
Neckera complanata		7	1	1	2	10
Polytrichum			1			1
Spahgnum affine			1			
Sphagnum indet.	1					
indet. sp.						2
