# Supplementary material for: Seventy-five mosses and liverworts found frozen with the late Neolithic Tyrolean Iceman: Origins, taphonomy and the Iceman’s last journey
Source: PLoS One. 2019 Oct 30;14(10):e0223752. doi: 10.1371/journal.pone.0223752 (PMC6821077; doi:10.1371/journal.pone.0223752)
Supplement: S5 Appendix — (PDF) [file pone.0223752.s005.pdf]

S5 Appendix. Mosses recovered from Caprine Faeces

The Martin Busch Hut lies at 2,500m in Niedertal which joins Ventertal at Vent. The area is grazed seasonally by many sheep and a few goats.

Mosses	Martin Busch Hut caprine droppings	Ötzi site samples caprine droppings
Bryum cf *pseudotriquetrum*	1	
Dicranum sp	1	
Heterocladium dimorphum		1
Isopterygiopsis pulchellum	1	
Polytichum piliferum	1	1
Racomitrium canescens sensu lato	2	
Racomitrium/Grimmia		1
Tortella cf *fragilis*	1	
Mosses indet.	1	2

In the Basle University laboratory of Professor Stefani Jacomet caprine droppings have been examined from many prehistoric sites in Switzerland

(including Arbon Bleiche 3; [47]) and a few from Germany and Italy. Moss remains were found frequently but never in large amount.
